# Assessing the impact of efficacy stopping rules on the error rates under the multi-arm multi-stage framework

**DOI:** 10.1177/1740774518823551

**Published:** 2019-01-16

**Authors:** Alexandra Blenkinsop, Mahesh KB Parmar, Babak Choodari-Oskooei

**Affiliations:** 1 MRC Clinical Trials Unit at UCL, London, UK

**Keywords:** Multi-arm, multi-stage, lack-of-benefit boundary, efficacy stopping boundary, familywise error rate, multi-arm multi-stage

## Abstract

**Background:**

The multi-arm multi-stage framework uses intermediate outcomes to assess lack-of-benefit of research arms at interim stages in randomised trials with time-to-event outcomes. However, the design lacks formal methods to evaluate early evidence of overwhelming efficacy on the definitive outcome measure. We explore the operating characteristics of this extension to the multi-arm multi-stage design and how to control the pairwise and familywise type I error rate. Using real examples and the updated nstage program, we demonstrate how such a design can be developed in practice.

**Methods:**

We used the Dunnett approach for assessing treatment arms when conducting comprehensive simulation studies to evaluate the familywise error rate, with and without interim efficacy looks on the definitive outcome measure, at the same time as the planned lack-of-benefit interim analyses on the intermediate outcome measure. We studied the effect of the timing of interim analyses, allocation ratio, lack-of-benefit boundaries, efficacy rule, number of stages and research arms on the operating characteristics of the design when efficacy stopping boundaries are incorporated. Methods for controlling the familywise error rate with efficacy looks were also addressed.

**Results:**

Incorporating Haybittle–Peto stopping boundaries on the definitive outcome at the interim analyses will not inflate the familywise error rate in a multi-arm design with two stages. However, this rule is conservative; in general, more liberal stopping boundaries can be used with minimal impact on the familywise error rate. Efficacy bounds in trials with three or more stages using an intermediate outcome may inflate the familywise error rate, but we show how to maintain strong control.

**Conclusion:**

The multi-arm multi-stage design allows stopping for both lack-of-benefit on the intermediate outcome and efficacy on the definitive outcome at the interim stages. We provide guidelines on how to control the familywise error rate when efficacy boundaries are implemented in practice.

## Introduction

The multi-arm multi-stage (MAMS) adaptive clinical trial design developed by Royston et al.^1,2^ has many practical advantages when evaluating treatments, such as increased efficiencies in time and patients required, and a greater probability of success than a traditional parallel-group, single-stage design.^3^ Interim stages are used to identify early evidence of lack-of-benefit of each research arm over the control arm. The MAMS framework utilises an intermediate (I) outcome measure for interim assessment, which is correlated with the definitive (D) primary outcome measure but may be observed earlier. The *I*-outcome may be composite, including the *D*-outcome. In time-to-event settings, the events required to trigger the interim analyses are accrued more quickly on the *I*-outcome, so decisions to drop arms for lack-of-benefit can be made earlier if I≠D than if the primary outcome is used throughout (I=D) . While other MAMS designs have been proposed, the framework considered here is unique in its application to time-to-event outcomes. The design has been implemented successfully in several randomised trials, some of which have been described in a recent article in Clinical Trials.^4^

Efficacy stopping boundaries can be implemented as a means of assessing interim data as they accumulate to identify treatment arms indicating overwhelming efficacy over the course of the trial. Data monitoring committees may recommend terminating a trial before its planned end in order to report the results, or be submitted for regulatory approval, earlier than planned. Permitting early stopping for efficacy would increase the efficiency of the MAMS design further, by minimising patients being exposed to inferior treatment regimens and decreasing the time for effective treatments to reach patients. Popular stopping boundaries are the Haybittle–Peto rule,^5^ the O’Brien–Fleming rule^6^ and other approaches utilising an alpha-spending function.^7,8^

Multiple testing in MAMS trials may increase the risk of a type I error,^9^ also known as the pairwise error rate (PWER) in two-arm designs. In a multi-arm setting, it is generally referred to as the familywise error rate (FWER): the probability of at least one ineffective research arm being recommended at an interim stage or at the end of the trial. Whether or not the FWER should be controlled in a MAMS trial should be decided on a case-by-case basis. However, it may be important that its value is calculated, even in trials that do not require strong control of the FWER.^10^

As far as we are aware, no alternative MAMS trial design formally assesses lack-of-benefit on an intermediate outcome and efficacy on the definitive outcome simultaneously at interim analyses for time-to-event data. For this reason, this extension to the existing framework of Royston and colleagues,^11,12^ and the development of the associated nstage software, will provide the necessary evidence required by regulatory agencies to allow interim efficacy guidelines to be incorporated into MAMS designs and allow trials to measure and control the impact on the operating characteristics of the design.

This article explores this design extension via a simulation study, to quantify the extent to which the error rates are affected by formal interim efficacy looks according to different design parameters. We also illustrate how the FWER can be controlled in practice by modifying the design specification, using real MAMS trials as examples.

### MAMS in practice: the STAMPEDE trial

Table 1 illustrates how the MAMS proposal has been applied to a clinical trial evaluating systemic therapies in prostate cancer.^13^ STAMPEDE was initially designed as a six-arm, four-stage trial, using the composite intermediate outcome measure of failure-free survival (FFS), for assessing lack-of-benefit at interim stages, and a definitive outcome of overall survival (OS) at the final analysis for efficacy. Table 1 shows the design specification for the original treatment comparisons at each stage: the outcome measure, target hazard ratio (HR) under the alternative hypothesis for the research arms (HR^1^), power $\left(\omega_{j}\right)$ , significance level $\left(\alpha_{j}\right)$ , critical HR to drop arms for lack-of-benefit and control arm events required to trigger each analysis. All *p*-values are one sided.

**Table 1.** Design specification for the six-arm four-stage STAMPEDE trial. $HR^{1}$ is the target hazard ratio; $\omega_{j}$ and $\alpha_{j}$ are the power and significance level, respectively, for stage *j*.

Stage (*j*)	Type	Outcome measure	HR^1^	$\omega_{j}\;(\%)$	$\alpha_{j}$	Critical HR	Control arm events
1	Activity	FFS	0.75	95	0.50	1.0	113
2	Activity	FFS	0.75	95	0.25	0.92	216
3	Activity	FFS	0.75	95	0.10	0.89	334
4	Efficacy	OS	0.75	90	0.025	–	403

HR: hazard ratio; FFS: failure-free survival; OS: overall survival.

## Methods

### The MAMS design

For a *K*-arm, *J*-stage trial, one-sided significance levels are specified for stages 1 to *J* to compare each of the research arms against the control arm on the intermediate outcome at interim analyses and definitive outcome at the final analysis. No formal comparisons are made between the research arms. The design targets high pairwise power at interim stages (e.g. 95%) to increase the probability of continuing with promising research arms.^2^ For the chosen power, the stagewise significance levels form a boundary for lack-of-benefit, since rejection of the null hypothesis at an interim analysis indicates that the arm continues recruitment to the subsequent stage.

The timing of interim analyses is driven by the number of intermediate outcome events observed in the control arm for trials with time-to-event outcomes and is determined by how liberal or conservative the significance levels are. Large *p*-values indicate early interim analyses, requiring only a small number of events. More conservative boundaries, with smaller *p*-values, trigger relatively later interim analyses when more events have been accrued. At each interim analysis, research arms demonstrating lack-of-benefit on the intermediate outcome may be dropped from the subsequent stages, optimising resources in the ongoing trial. By allowing the specification of an efficacy boundary, recruitment can also be terminated early to the research arms demonstrating overwhelming evidence of efficacy on the definitive outcome at an interim analysis. Detailed guidelines for designing a MAMS trial are provided in Supplemental Appendix A.

In the MAMS design, correlation is induced between the estimated treatment effects of pairwise comparisons in two ways: first due to the shared control arm and second due to the shared or correlated outcome measures across stages. In the case of STAMPEDE, the intermediate and definitive outcome measures were strongly correlated due to FFS being a composite measure of OS (see Supplemental Appendix C), but the source of the correlation may differ for alternative outcome measures.

### Type I error rate

In the MAMS setting, type I errors can only be made on decisions based on the definitive outcome. The PWER for comparison *k* is defined as the probability of a type I error made on comparison *k*, while the FWER is the probability of a type I error made on any pairwise comparison.

For a design where I=D , assuming that lack-of-benefit boundaries are binding, the PWER is conditional on the probability of treatment arms not being dropped for lack-of-benefit at previous stages. When efficacy boundaries are implemented, the PWER can be calculated as follows

(1) $$\begin{array}{c} \mathrm{PWER}=\mathrm{Pr}(Z_{1k}<b_{1})+\int_{b_{1}}^{l_{1}}\int_{-\infty }^{b_{2}}f\left(z_{1k},z_{2k};\Sigma_{2}|H_{0}^{k}\right)dz_{2k}dz_{1k}\\ \;\;\;+\cdots \\ \;\;\;+\int_{b_{1}}^{l_{1}}\cdots \int_{b_{J-1}}^{l_{J-1}}\int_{-\infty }^{b_{J}}f\left(z_{1k},\ldots ,z_{(J-1)k},z_{\mathrm{Jk}};\Sigma_{J}|H_{0}^{k}\right)\\ \;\;\;dz_{\mathrm{Jk}}dz_{(J-1)k}\ldots dz_{1k} \end{array}$$

where $\left(z_{1k},\ldots ,z_{\mathrm{Jk}}\right)$ is a realisation of the $\left(Z_{1k},\ldots ,Z_{\mathrm{Jk}}\right)$ test statistics comparing the experimental arm k=1,…,K against the control arm at stage j=1,…,J , with each $Z_{\mathrm{jk}}$ following a standard normal distribution; $l_{1},\ldots ,l_{J}$ are the upper boundaries for lack-of-benefit and $b_{1},\ldots ,b_{J}$ are the lower bounds for efficacy in the time-to-event setting and $\Sigma_{2},\ldots ,\Sigma_{J}$ are the correlation matrices under the null hypothesis for the kth comparison, $H_{0}^{k}$ (see Supplemental Appendix C for an example).

To calculate the FWER, the union of all events leading to a type I error is considered. The probability also depends on whether the trial continues with the remaining arms or is terminated when a research arm crosses the efficacy boundary. In the former case, when I=D , the FWER is calculated by considering all permutations of type I errors possible across the pairwise comparisons under the assumption that all research arms are ineffective on *D* (the global null).^14^ For the latter scenario, an analytical solution has been derived.^15,16^ We compared these two approaches empirically by simulation.

In cases where an intermediate outcome measure is used for assessing lack-of-benefit at interim (I≠D) , the maximum PWER is considered. This measure assumes that each research arm appears sufficiently effective on *I* to pass all lack-of-benefit assessments under the global null on *D*. Thus, the lack-of-benefit stopping boundaries are considered non-binding; hence, where no efficacy looks are permitted, it has been shown that the maximum PWER is equal to the final-stage significance level of the design $\left(\alpha_{J}\right)$ .^17^ When efficacy bounds are implemented on the definitive outcome, it is equal to the probability of a type I error made at an interim or the final stage. The maximum FWER for a design with efficacy bounds can correspondingly be evaluated by considering all permutations of type I errors across the pairwise comparisons, assuming non-binding lack-of-benefit bounds. Analytical solutions can be obtained as above, by calculating the correlation structure ∑ and replacing the lack-of-benefit bounds with infinity.

### Power

The power of a clinical trial is the probability an effective treatment is identified by the final analysis. In the MAMS setting, assuming binding boundaries, the power is conditional on the treatment arm passing all interim stages prior to rejection of the null hypothesis, without being dropped for lack-of-benefit. Three different definitions of power can be calculated in multi-arm trials: per-pair, any-pair and all-pair powers.^18^ Per-pair power is the probability of detecting a treatment effect in a particular arm. Any-pair power is the probability of detecting at least one true treatment effect among several arms and all-pair power is the probability of detecting every true treatment effect from all pairwise comparisons. The measures are calculated under the global alternative hypothesis: the assumption that all research arms are effective. The three measures of power will be identical in a two-arm trial,^19^ but when considering a multi-arm design the power measure of interest may depend on the objective of the trial. When efficacy bounds are implemented, per-pair power can be evaluated using a generalised form of equation (1) under the alternative hypothesis $H_{A}$ (see Supplemental Appendix B).

### Simulation study

Treatment arm–level data were simulated for 3 million trials. The type I error measure of interest was the PWER for two-arm scenarios and the FWER for multi-arm settings. Multi-arm scenarios considered the three measures of powers previously defined in section ‘Power’. Operating characteristics were evaluated empirically from the simulation results, though were compared against analytical solutions for the two-arm scenarios.

We explored the impact of implementing efficacy stopping rules on the type I error and power under different plausible design specifications which may be implemented in a MAMS trial, as described below. A *separate stopping* rule^20^ was assumed for all the results presented, with operating characteristics calculated assuming that the trial continues with the remaining research arms if any one arm is dropped early for efficacy. However, the impact of terminating the trial after this occurrence was also investigated, since in some cases it may be unethical to continue the trial. This approach to stopping early for efficacy has been termed a *simultaneous stopping* rule.

Simulations under an I≠D design assume non-binding lack-of-benefit boundaries, but both binding and non-binding stopping rules were considered when I=D to explore the sensitivity of the results to the assumption of binding boundaries.

### Definition of simulation parameters

#### Efficacy stopping rule

The form of the efficacy stopping rule will determine how stringent the boundaries $\alpha_{E1},\ldots ,\alpha_{\mathrm{EJ}}$ are. A three-stage design was used to examine the impact of varying the first- and second-stage efficacy bounds, where the third bound was fixed at the final-stage significance level to ensure a meaningful conclusion to the trial $\left(\alpha_{\mathrm{EJ}}=\alpha_{J}=0.025\right)$ .

Assuming survival outcomes, only beneficial treatment effects were considered (i.e. HR < 1) so the lack-of-benefit thresholds serve as an upper boundary and the efficacy thresholds as a lower boundary. The direction of these may differ for alternative outcomes.

The Haybittle–Peto guideline^5^ uses the same threshold at stages 1 to J−1 . Under this guideline, the simulations required a one-sided *p*-value of 0.0005 to declare overwhelming efficacy at interim for a treatment comparison on the *D*-outcome. The O’Brien–Fleming guideline^6^ adjusts the threshold at each stage required to declare efficacy in order to control the overall probability of a type I error at a prespecified level. It is based on the information time $t^{*}$ : the proportion of events observed by interim analysis *j* out of the total expected by the final-stage analysis. An alpha-spending function to approximate the O’Brien–Fleming boundary was provided by DeMets and Lan.^21^ We also explored the impact of ‘custom’ efficacy boundaries, which allow greater flexibility in how liberal or conservative each interim assessment should be in order to stop early for efficacy.

The Haybittle–Peto guideline was used as a default rule when investigating other design parameters, since it is unaffected by the timing of the stages.

#### Other design parameters

Table 2 shows the range of values used in the simulation study, for the parameters known to have an influence on the operating characteristics of the MAMS design. The times at which the interim analyses are to be conducted are dictated by the stagewise significance levels for assessing lack-of-benefit. A large $\alpha_{j}$ is recommended in the first stage to trigger an early interim analysis for lack-of-benefit while retaining high power, with the function $\alpha_{j}=0.5^{j}$ (j=1,…,J) suggested by Royston et al.^2^

**Table 2.** Simulation parameter values.

Design parameter	Simulation inputs
Number of comparisons	1, 2, 3, 4, 5
Number of stages	2, 3, 4
Allocation ratio	0.5, 0.6, 0.7, 0.8, 0.9, 1
Final-stage significance level	0.01, 0.025, 0.05
$\alpha_{1}$	0.1, 0.2, 0.3, 0.4, 0.5
Outcome measures	I=D , I≠D
Number of simulations	3,000,000

We used one-sided lack-of-benefit boundaries of α=(0.1,0.025) for the two-stage, α=(0.25,0.1,0.025) for the three-stage and α=(0.5,0.25,0.1,0.025) for the four-stage simulations. The default value for the final-stage significance level was fixed at $\alpha_{J}=0.025$ for all simulations, to reflect the conventional test for assessing efficacy, for example, in the STAMPEDE trial.^13^ We carried out simulations under both I=D and I≠D designs, where *I* is FFS with a HR of 1 under the null and 0.70 under the alternative and *D* is OS with a HR of 1 under the null and 0.75 under the alternative. The strength of correlation between the treatment effects on *I* and *D* does not strongly influence the maximum error rates, since non-binding boundaries are assumed when an intermediate outcome is used to assess lack-of-benefit, and type I errors can only be made on the definitive outcome.

### Strong control of the FWER

Controlling the FWER in the strong sense limits its value under any underlying treatment effect of the *I*- or *D*-outcomes. The maximum FWER is calculated by assuming non-binding lack-of-benefit boundaries, such that all research arms pass all interim stages. The actual FWER of the trial must be less than or equal to this maximum and so it is controlled.

A program was written using linear interpolation to determine the final-stage significance level $\left(\alpha_{J}\right)$ required to strongly control the FWER at the prespecified level. This was run both with and without efficacy boundaries and the designs were compared to their original specifications. The incremental adjustment to $\alpha_{J}$ and the additional control arm events required with the implementation of efficacy bounds to the design was measured.

We applied this method to two MAMS trials utilising an intermediate outcome measure (i.e. I≠D ): ICON5^22^ and STAMPEDE.^3,13^ Both trials aimed to control the PWER instead of the FWER, since the research questions in each pairwise comparison were assumed distinct at the time of the design. However, we show how strong control of the FWER at 2.5% (one-sided) could have been achieved.

## Results

### Simulation results

#### Two-arm designs

Our simulations indicate that in a two-arm two-stage design the inclusion of the Haybittle–Peto efficacy rule at the interim stage has a minimal impact on the PWER under any configuration of the timing of interim analysis, the value of the final-stage significance level and the design allocation ratio. See Supplemental Appendix E for further details of these results. The extent of inflation of the FWER is determined by the choice of efficacy stopping boundary and whether an intermediate outcome is used (see Table 3). While non-binding lack-of-benefit boundaries increase the absolute FWER, the relative inflation is no larger than that under binding boundaries so the assumed approach does not affect the interpretation of the results presented.

**Table 3.** Impact of the choice of efficacy boundary (EB) $\alpha_{E1},\ldots ,\alpha_{E3}$ on the type I error rate (all SEs < 0.0002; lack-of-benefit boundaries = 0.25, 0.1, 0.025; allocation ratio = 1).

					Type I error rate				Power	
	Rule	$\alpha_{E1}$	$\alpha_{E2}$	$\alpha_{E3}$	No EB	With EB	Inflation	Percentage	No EB	With EB
I=D , binding	Peto	0.0005	0.0005	0.0250	0.0224	0.0225	0.0001	0	0.8771	0.8771
	Custom	0.0005	0.0010	0.0250	0.0224	0.0225	0.0001	0	0.8771	0.8771
	Custom	0.0005	0.0020	0.0250	0.0224	0.0226	0.0002	1	0.8771	0.8771
	Custom	0.0005	0.0050	0.0250	0.0224	0.0229	0.0005	2	0.8771	0.8771
	Custom	0.0005	0.0100	0.0250	0.0224	0.0242	0.0018	8	0.8771	0.8771
	Custom	0.0010	0.0010	0.0250	0.0224	0.0227	0.0003	1	0.8771	0.8771
	Custom	0.0010	0.0020	0.0250	0.0224	0.0227	0.0003	1	0.8771	0.8771
	Custom	0.0010	0.0050	0.0250	0.0224	0.0230	0.0006	3	0.8771	0.8771
	Custom	0.0010	0.0100	0.0250	0.0224	0.0243	0.0019	8	0.8771	0.8771
	O’Brien	0.0022	0.0139	0.0250	0.0224	0.0261	0.0037	17	0.8771	0.8771
I=D , non-binding	Peto	0.0005	0.0005	0.0250	0.0250	0.0250	0.0000	0	0.8999	0.8999
	Custom	0.0005	0.0010	0.0250	0.0250	0.0250	0.0000	0	0.8999	0.8999
	Custom	0.0005	0.0020	0.0250	0.0250	0.0251	0.0001	0	0.8999	0.8999
	Custom	0.0005	0.0050	0.0250	0.0250	0.0254	0.0004	2	0.8999	0.8999
	Custom	0.0005	0.0100	0.0250	0.0250	0.0267	0.0017	7	0.8999	0.8999
	Custom	0.0010	0.0010	0.0250	0.0250	0.0252	0.0002	1	0.8999	0.8999
	Custom	0.0010	0.0020	0.0250	0.0250	0.0255	0.0002	1	0.8999	0.8999
	Custom	0.0010	0.0050	0.0250	0.0250	0.0268	0.0005	2	0.8999	0.8999
	Custom	0.0010	0.0100	0.0250	0.0250	0.0287	0.0018	7	0.8999	0.8999
	O’Brien	0.0022	0.0139	0.0250	0.0250	0.0282	0.0037	13	0.8999	0.8999
I≠D , non-binding	Peto	0.0005	0.0005	0.0250	0.0250	0.0255	0.0005	2	0.9002	0.9002
	Custom	0.0005	0.0010	0.0250	0.0250	0.0258	0.0008	3	0.9002	0.9002
	Custom	0.0005	0.0020	0.0250	0.0250	0.0264	0.0014	6	0.9002	0.9002
	Custom	0.0005	0.0050	0.0250	0.0250	0.0285	0.0035	14	0.9002	0.9002
	Custom	0.0005	0.0100	0.0250	0.0250	0.0323	0.0073	29	0.9002	0.9002
	Custom	0.0010	0.0010	0.0250	0.0250	0.0261	0.0011	4	0.9002	0.9002
	Custom	0.0010	0.0020	0.0250	0.0250	0.0267	0.0017	7	0.9002	0.9002
	Custom	0.0010	0.0050	0.0250	0.0250	0.0287	0.0037	15	0.9002	0.9002
	Custom	0.0010	0.0100	0.0250	0.0250	0.0324	0.0074	30	0.9002	0.9002
	O’Brien	<0.0001	0.0001	0.0250	0.0250	0.0250	0.0000	0	0.9002	0.9002

Implementing the Haybittle–Peto rule in a three-stage design ( $\alpha_{\mathrm{Ej}}=0.0005$ at each interim stage) inflates the FWER by less than 1% when outcomes *I* and *D* are equal and the maximum FWER by 2% when different, so it can be implemented with minimal penalty on the type I error in both settings. Less conservative rules may result in larger inflation of the error rates, illustrated in Table 3.

For a design where *I* = *D* and $\alpha_{E1}=0.0005$ , custom second-stage efficacy bounds of $\alpha_{E2}=0.001\;\mathrm{or}\;0.002$ have no impact on the type I error rate. Increasing $\alpha_{E2}$ to 0.005 and 0.01 shows the FWER may be inflated by 2% and 8%, respectively, suggesting that a custom second-stage boundary can increase the Haybittle–Peto bound 10-fold with minimal impact on the FWER. Increasing $\alpha_{E1}$ to 0.001 only inflates the FWER with liberal second-stage efficacy boundaries $\left(\alpha_{E2}>0.005\right)$ , suggesting that the efficacy bound at the first interim analysis can be less conservative than the Haybittle–Peto bound. When I≠D , the inflation is much larger than that when *I* = *D* where custom boundaries are used with a liberal second-stage *p*-value, with the FWER inflated by up to 15% when $\alpha_{E2}=0.005$ and by almost a third when $\alpha_{E2}=0.01$ $\left(\alpha_{E1}\leq 0.001\right)$ .

An O’Brien–Fleming type rule inflates the FWER the most by 17% when *I* = *D*, due to the liberal *p*-values required at the first two stages to declare efficacy (e.g. $\alpha_{E2}=0.0139$ ). When I≠D , this is the only rule where the inflation of the FWER is smaller than that when *I* = *D*, due to the *I*-outcome measure allowing the interim analyses to occur much earlier with a smaller number of *D*-events. Thus, the spending function requires very small *p*-values (<0.0001) at the early interim stages to declare efficacy, and as such no inflation of the maximum FWER is incurred under this trial design when I≠D .

#### MAMS designs

Table 4 shows the impact of increasing the number of pairwise comparisons and stages when *I* = *D* and I≠D assuming binding and non-binding lack-of-benefit boundaries, respectively. Increasing the number of pairwise comparisons in a two-stage design incurs no inflation of the FWER when *I* = *D* and the relative inflation remains below 2% when I≠D .

**Table 4.** Impact of the number of stages and arms on the FWER with Haybittle–Peto efficacy boundary (EB; *p* = 0.0005) (all SEs < 0.0002; lack-of-benefit boundaries as described in text; allocation ratio = 1 (for alternative allocation ratios in two-stage designs, see Supplemental Appendix E)).

			FWER				Per-pair power		Any-pair power		All-pair power	
Comparisons		Stages	No EB	With EB	Inflation	Percentage	No EB	With EB	No EB	With EB	No EB	With EB
*I* = *D*, binding	1	2	0.0239	0.0240	0.0001	0	0.8940	0.8940	0.8940	0.8940	0.8940	0.8940
		3	0.0224	0.0225	0.0001	0	0.8771	0.8771	0.8771	0.8771	0.8771	0.8771
		4	0.0213	0.0217	0.0004	2	0.8553	0.8553	0.8553	0.8553	0.8553	0.8553
	2	2	0.0437	0.0437	0.0000	0	0.8942	0.8942	0.9650	0.9650	0.8234	0.8234
		3	0.0410	0.0412	0.0002	0	0.8773	0.8773	0.9575	0.9575	0.7971	0.7971
		4	0.0391	0.0397	0.0006	2	0.8554	0.8554	0.9475	0.9475	0.7634	0.7634
	3	2	0.0605	0.0605	0.0000	0	0.8941	0.8941	0.9830	0.9830	0.7705	0.7705
		3	0.0570	0.0572	0.0002	0	0.8772	0.8772	0.9788	0.9788	0.7380	0.7380
		4	0.0543	0.0552	0.0009	2	0.8554	0.8554	0.9731	0.9732	0.6971	0.6971
	4	2	0.0752	0.0752	0.0000	0	0.8940	0.8940	0.9900	0.9900	0.7283	0.7283
		3	0.0708	0.0711	0.0003	0	0.8769	0.8769	0.9873	0.9873	0.6912	0.6912
		4	0.0677	0.0688	0.0011	2	0.8552	0.8552	0.9837	0.9837	0.6458	0.6458
	5	2	0.0882	0.0882	0.0000	0	0.8939	0.8939	0.9934	0.9934	0.6934	0.6934
		3	0.0833	0.0837	0.0004	0	0.8769	0.8769	0.9915	0.9915	0.6537	0.6537
		4	0.0798	0.0811	0.0013	2	0.8553	0.8553	0.9891	0.9891	0.6049	0.6049
I≠D , non-binding	1	2	0.0250	0.0253	0.0003	1	0.9001	0.9001	0.9001	0.9001	0.9001	0.9001
		3	0.0250	0.0255	0.0005	2	0.9002	0.9002	0.9002	0.9002	0.9002	0.9002
		4	0.0250	0.0260	0.0010	4	0.9000	0.9000	0.9000	0.9000	0.9000	0.9000
	2	2	0.0455	0.0460	0.0005	1	0.9001	0.9001	0.9677	0.9677	0.8326	0.8326
		3	0.0455	0.0463	0.0008	2	0.9002	0.9002	0.9676	0.9676	0.8327	0.8327
		4	0.0455	0.0472	0.0017	4	0.9000	0.9000	0.9676	0.9676	0.8325	0.8325
	3	2	0.0628	0.0635	0.0007	1	0.9001	0.9001	0.9845	0.9845	0.7818	0.7818
		3	0.0627	0.0644	0.0017	3	0.9001	0.9001	0.9843	0.9843	0.7818	0.7818
		4	0.0627	0.0649	0.0022	4	0.9001	0.9001	0.9845	0.9845	0.7816	0.7816
	4	2	0.0780	0.0792	0.0012	2	0.9001	0.9001	0.9909	0.9909	0.7413	0.7413
		3	0.0780	0.0798	0.0018	2	0.9000	0.9000	0.9909	0.9909	0.7412	0.7412
		4	0.0780	0.0809	0.0029	4	0.9000	0.9000	0.9910	0.9910	0.7410	0.7410
	5	2	0.0916	0.0927	0.0011	1	0.9000	0.9000	0.9941	0.9941	0.7076	0.7076
		3	0.0915	0.0938	0.0023	3	0.9000	0.9000	0.9940	0.9940	0.7079	0.7079
		4	0.0915	0.0950	0.0035	4	0.9000	0.9000	0.9941	0.9941	0.7076	0.7077

FWER: familywise error rate.

The relative inflation increases with the number of stages in the trial, as the number of opportunities to drop arms early for efficacy increases. However, the inflation when *I* = *D* is arguably negligible at less than 2%, and the maximum FWER inflation remains below 5% when I≠D for a trial with up to four stages.

Extending the design to MAMS settings does not materially change the results observed from the two-arm two-stage simulations. While the absolute FWER naturally increases, there is no impact on the relative effect of incorporating efficacy looks with more research arms, and the relative inflation when increasing stages remains constant with any number of arms.

In accordance with Table 3, an O’Brien–Fleming type rule implemented in a MAMS design inflates the FWER by up to 17% when *I* = *D*, but no inflation of the maximum FWER is observed when I≠D (see Supplemental Appendix E).

The three power measures are almost unaffected by the implementation of efficacy boundaries for all possible design configurations. The induced between-arm correlation due to the common control arm is found to increase all-pair power, compared to a design with independent treatment arms, and (negligibly) decrease any-pair power.

When adopting a simultaneous stopping rule, the FWER is unaffected by whether or not the trial terminates early compared to a separate stopping rule. Since the FWER measures the probability of at least one type I error under the global null, type I errors made after an arm is dropped for efficacy do not increase the FWER. Simulations found that the PWER decreases marginally (e.g. by 0.001 for a four-stage design with four arms).

### Example: implementing efficacy boundaries in MAMS trials

The operating characteristics for the example MAMS trials STAMPEDE and ICON5 are shown in Table 5 for the original design specifications and with each of the three efficacy stopping rules. Both trials observe some inflation of the type I error when efficacy bounds are hypothetically incorporated, due to the use of an intermediate outcome, reflecting the theoretical results observed in the simulation study. How to control the FWER in these trials for such stopping rules is also demonstrated.

**Table 5.** Impact on operating characteristics of STAMPEDE and ICON5 when controlling the FWER at 2.5% with the addition of efficacy boundaries (EBs). The designs with no EBs assessed non-binding lack-of-benefit only at interim analyses (ICON5: α=0.064,0.025 , STAMPEDE: 0.5,0.25,0.1,0.025 ).

		No FWER control				FWER controlled at 2.5%			
Example trial	Measure	No EB	HP EB (*p* = 0.0005)	OBF EB	Custom EB^a^	No EB	HP EB (*p* = 0.0005)	OBF EB	Custom EB^a^
ICON5	$\alpha_{J}$	0.025	0.025	0.025	0.025	0.0073	0.0069	0.0073	0.0064
	Control arm events	424	424	424	424	527	532	527	538
	Power	0.98	0.98	0.98	0.98	0.98	0.98	0.98	0.98
	PWER	0.0250	0.0251	0.0251	0.0256	0.0073	0.0073	0.0073	0.0072
	FWER	0.0781^b^	0.0782	0.0781	0.0798	0.0250	0.0250	0.0250	0.0250
STAMPEDE	$\alpha_{J}$	0.025	0.025	0.025	0.025	0.0055	0.0043	0.0055	0.0026
	Control arm events	403	403	403	403	555	579	555	626
	Power	0.90	0.90	0.90	0.90	0.90	0.90	0.90	0.90
	PWER	0.0250	0.0257	0.0252	0.0266	0.0055	0.0054	0.0055	0.0054
	FWER	0.1032^b^	0.1059	0.1039	0.1093	0.0250	0.0250	0.0250	0.0250

FWER: familywise error rate; HP: Haybittle–Peto; OBF: O’Brien–Fleming; PWER: pairwise error rate.

a ICON5: $\alpha_{\mathrm{Ej}}=0.001$ , j=1 STAMPEDE: $\alpha_{\mathrm{Ej}}=0.0005,0.001,0.002$ , j=1,2,3 .

b The actual FWER in both trials differed due to the research arms being dropped, as described in the text.

The two-stage ICON5 trial, when retrospectively designed with the Haybittle–Peto stopping rule, would require the final-stage significance level $\alpha_{J}$ to be reduced minimally by 0.0004, with only five (<1%) additional control arm events to be observed, in order to maintain the same level of FWER control as only assessing for lack-of-benefit. The O’Brien–Fleming type rule can be implemented without any further adjustment to $\alpha_{J}$ , but the probability of dropping arms early for efficacy is very low at interim (<0.0001). Controlling the FWER with a ‘custom’ efficacy boundary of $\alpha_{E}=0.001$ at the interim analysis would require 2% more control arm events, and the greatest reduction in $\alpha_{J}$ of the three rules to 0.0064, but in general settings the degree of adjustment will depend on the specific custom boundary used. Note that recruitment to ICON5 was discontinued at the interim analysis following the first stage, since no research arm passed the lack-of-benefit assessment on progression-free survival.

For the original STAMPEDE design, the trial would be vulnerable to greater inflation than ICON5 when incorporating efficacy bounds at interim for the definitive outcome, due to the additional two stages in the design. A total of 19 (3%) additional control arm events would be required to control the maximum FWER at 2.5% when using a Haybittle–Peto efficacy stopping rule compared to a design only assessing lack-of-benefit, reducing $\alpha_{J}$ from 0.0055 to 0.0045. The O’Brien–Fleming type boundary controls the FWER at 2.5% without additional adjustment to $\alpha_{J}$ . As shown by the simulation study, the stopping rule is too conservative to impact the type I error rate, due to the use of an intermediate outcome measure for lack-of-benefit assessment. A custom interim rule of *α_E_* = (0.0005, 0.001, 0.002) requires the most extreme modification to the design in order to control the FWER, with an $\alpha_{J}$ of 0.0027 requiring 12% more control arm events to be accrued in order to have the designed power to test at this significance level. For the original comparisons in the STAMPEDE trial, two research arms were dropped for insufficient benefit during the trial; as such, the actual FWER for the remaining arms was 6.75%, not 10.32% as reported in Table 5.

## Discussion

In this article, we have demonstrated how efficacy stopping rules can be incorporated into MAMS designs under the framework of Royston et al. We have also addressed concerns about how the operating characteristics would be affected by early assessments for efficacy on the definitive outcome. There is no consensus under which circumstances the FWER should be controlled.^10,23^ However, we have demonstrated how to control the FWER in practice if required, using the four-stage original STAMPEDE trial design as an example, by modifying the final-stage significance level, thereby increasing the number of patients and length of the trial. Control of the PWER could be achieved using the same methods by specifying the trials as two-arm designs.

In summary, our findings suggest that (binding) lack-of-benefit stopping rules will generally decrease the type I error rates and, marginally, the power. In contrast, efficacy stopping boundaries have the potential to increase the type I error rate with no impact on power. The simulation results indicate that the extent of this increase primarily depends on the shape and *p*-value thresholds of the stopping rule used. They also show that in two-stage designs the inflation remains below 2% for varying configurations of the allocation ratio, number of research arms and timing of the analyses. Designs with three or more stages may see greater inflation of the FWER when I≠D . Other parameters with a stronger influence on the impact of efficacy looks on the FWER are the use of an intermediate outcome measure and the final-stage significance level.

**Figure 1.**
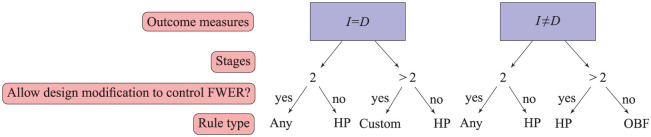
Choosing an efficacy stopping rule based on the design and willingness to modify $\alpha_{J}$ to control FWER. HP is the Haybittle–Peto rule (*p* = 0.0005) and OBF is an O’Brien–Fleming type rule. ‘Any’ indicates that the design is not vulnerable to inflation, so the rule used can be flexible; ‘custom’ indicates that a more liberal boundary than Haybittle–Peto can be applied.

When choosing an efficacy stopping boundary, for a three-stage design the Haybittle–Peto rule was not observed to inflate the FWER but can be conservative. When I≠D , the Haybittle–Peto rule is recommended, but more liberal custom rules can be used without inflating the FWER when *I* = *D*. An O’Brien–Fleming type rule can be implemented in a trial when I≠D , without any adjustment of the trial design to control the maximum FWER compared to a design which controls the FWER with lack-of-benefit boundaries only. Such a rule, however, is extremely unlikely to drop arms early due to the very conservative threshold required to declare efficacy. For this reason, this rule is not recommended providing that the investigator is willing to modify the design as demonstrated in this article in order to control the FWER. Figure 1 can be used to assist in choosing an efficacy rule depending on the design specification and how flexible the design is to accommodate FWER control. Since non-binding lack-of-benefit boundaries are often a regulatory requirement, with calculation of an upper bound for the FWER ensuring that strong control can be achieved, we recommend efficacy boundaries be implemented under non-binding lack-of-benefit analysis. However, when there are resource restrictions, for example, where treatment selection occurs, it may be necessary for stopping boundaries to be binding.

A fundamental aspect of the design is that the timing of interim analyses is driven by the accrual of control arm events on the intermediate outcome. At the design stage, it should be considered whether it is too early to assess efficacy at the interim stages based on the number of events expected on the definitive outcome. If data from previous trials are available, a judgement can easily be made on whether or not to implement efficacy boundaries; otherwise, a sensitivity analysis can be made under different assumptions for the distribution of *I*- and *D*-outcomes. Royston et al.^2^ recommend the significance level for lack-of-benefit at stage 1 be no larger than 0.5 to ensure that an adequate number of events have been accrued, with STAMPEDE expecting 57 primary events on the control arm by the first interim analysis under the design assumptions.

Considering the use of hypothesis testing, early assessments of efficacy may result in some small bias in the point estimates for the arms dropped early. Choodari-Oskooei et al.^24^ demonstrated how bias in point estimates for arms dropped for lack-of-benefit is reduced by following up patients until the planned end of the trial. We expect to observe a similar result with efficacy boundaries, but this should be formally explored.

The choice and definition of error rates depend on the research question and the design of a MAMS trial. There are at least three possible approaches on how to proceed should a pairwise comparison for a research arm cross an efficacy boundary: (1) stop the trial and cease recruitment to all arms; (2) continue with the remaining research arms to make the final decision based on the totality of evidence and (3) add the efficacious regimen to the remaining arms and continue with combination therapies in both control and remaining research arms (e.g. the approach taken in STAMPEDE).^4^ Note that this is only appropriate where the original research arms include the control arm. The results in this article have investigated the first two approaches (focusing on the second), but can also handle the third, since pairwise comparisons are only made between the research and control arms on patients recruited contemporaneously. Some alternative MAMS designs adopt the first approach, where it may be of interest to stop the entire trial as soon as an effective regimen is identified, such as in dose-ranging trials. Examples of these are the MAMS design proposed by Magirr et al.^15^ using the MAMS package in R and the EAST6 software (http://www.cytel.com/software/east), though neither can accommodate intermediate measures for time-to-event outcomes at the time of submission.

We have updated the nstage program and help documentation in Stata to support the use of efficacy stopping rules in MAMS trial designs and the option to search for boundaries which preserve the FWER at the desired level assuming non-binding lack-of-benefit boundaries.^25^ The PWER, FWER and the three power measures described are evaluated by simulation in the program. See Supplemental Appendix D for the relevant commands.

Efficacy stopping rules can easily be implemented for alternative outcome measures in MAMS designs, such as binary or continuous outcomes, using the same principles applied here. The impact on the FWER can be investigated by following the same simulation procedure in nstage^12^ to evaluate the FWER.

## Supplemental Material

Supplementary_material – Supplemental material for Assessing the impact of efficacy stopping rules on the error rates under the multi-arm multi-stage framework Supplemental material, Supplementary_material for Assessing the impact of efficacy stopping rules on the error rates under the multi-arm multi-stage framework by Alexandra Blenkinsop, Mahesh KB Parmar and Babak Choodari-Oskooei in Clinical Trials
