# Supplementary material for: Assessing the impact of efficacy stopping rules on the error rates under the multi-arm multi-stage framework
Source: Clin Trials. 2019 Jan 16;16(2):132–41. doi: 10.1177/1740774518823551 (PMC6442021; doi:10.1177/1740774518823551)
Supplement: Supplementary_material – Supplemental material for Assessing the impact of efficacy stopping rules on the error rates under the multi-arm multi-stage framework [file Supplementary_material.pdf]

Appendix A Designing a MAMS trial

The steps for designing a MAMS trial implementing efficacy stopping boundaries with time-to-event outcomes are given below:

1. Choose the number of experimental arms, K, and stages, J.
2. Define the null values for the log hazard ratios on the intermediate and definitive outcomes, Δ_0^I, Δ_0^D.
3. Choose the allocation ratio A to be the number of patients allocated to each research arm for every patient allocated to the control arm.
4. Choose a significance level for lack-of-benefit and the target power for each stage (α_j, ω_j). These are one-sided since the design seeks to continue recruiting to arms which are performing no worse than the control. δ_{jk} is the critical value for rejecting the null hypothesis relating to α_j.
5. Choose an optional efficacy stopping boundary α_{Ej} for each stage $1, \ldots, J$, where $\alpha_{EJ} = \alpha_J$. δ_{Ejk} is the critical value for rejecting the null hypothesis relating to α_{Ej}.
6. Specify the minimum clinically relevant target hazard ratio on the intermediate and definitive outcomes, Δ_1^I, Δ_1^D.
7. Calculate the number of control arm events required to trigger each analysis, and the operating characteristics of the design. See Royston et al (2011) for sample size formulae and how to calculate trial timelines.
8. At each analysis $1, \ldots, J-1$, the treatment effects on I and D are estimated by $\widehat{\Delta}_{jk}^I$ and $\widehat{\Delta}_{jk}^D$ respectively, with p_{jk}^I and p_{jk}^D their corresponding p-values.
    - If $p_{jk}^D \leq \alpha_{Ej}$, reject the null hypothesis corresponding to the definitive outcome and claim efficacy.
    - If $p_{jk}^I \geq \alpha_{jk}$, the corresponding null hypothesis cannot be rejected and the recruitment to research arm k should be stopped for lack-of-benefit of k over the control arm.
    - If $p_{jk}^I < \alpha_{jk}$, continue recruitment to the next stage.
9. At the final analysis J, the treatment effect is estimated on D for each research arm, and one of two conclusions can be made:
    - If $p_{Jk}^D \leq \alpha_{Jk}$, reject the null hypothesis corresponding to the definitive outcome and claim efficacy.
    - If $p_{Jk}^D \geq \alpha_{Jk}$, the corresponding null hypothesis cannot be rejected at the α_J level.

Appendix B General formula for the pairwise error rates

$$PWER = P(Reject H_0^k|H_0^k) = \bigcup_{j=1}^{J}(Z_{jk} < b_j, b_1 < Z_{1k} < l_1, b_2 < Z_{2k} < l_2, \ldots, b_{j-1} < Z_{(j-1)k} < l_{j-1}|H_0^k)$$
$$= \sum_{j=1}^{J} \int_{b_1}^{l_1} \cdots \int_{-\infty}^{b_j} f((z_{1k}, \ldots, z_{jk}); \Sigma_j|H_0^k) dz_{jk} \ldots dz_{1k}$$

where $(z_{1k}, \ldots, z_{jk})$ is a realisation of the $(Z_{1k}, \ldots, Z_{Jk})$ and follows a multivariate normal distribution with mean Δ_{jk}^D and correlation matrix Σ, whose $(i,j)^{th}$ element is the between-stage correlation of treatment effects on the outcome measures in stage i and stage j $(i < j)$. H_0^k is the null hypothesis for comparison k, i.e. $\Delta_{jk}^D = 0$. When boundaries are non-binding, or when $I \neq D$, the $l_1, \ldots, l_{j-1}$ are set to ∞.

For calculation of the pairwise power, a similar formula applies under the alternative hypothesis, H_1^k:log(HR)=Δ_1^D, with the corresponding correlation matrix Σ under H_1^k.

Appendix C Example of correlation

The correlation matrix for the original STAMPEDE trial was estimated to be:

$$\Sigma_4 = \begin{bmatrix} 1 & 0.71 & 0.57 & 0.38 \\ 0.71 & 1 & 0.80 & 0.53 \\ 0.57 & 0.80 & 1 & 0.67 \\ 0.38 & 0.53 & 0.67 & 1 \end{bmatrix}$$

where each element Σ_{ij} is the correlation between the log hazard ratios at stages i and j $(j = 1, 2, 3, 4, i < j)$ on the definitive outcome, overall survival. The matrix for the correlation between the intermediate and definitive outcome measures for this design is included in Royston et al (2011).

Appendix D Stata commands

Below we provide an example of the Stata commands for running the updated `nstage` program with the option of three different efficacy stopping boundaries, which provides the simulated estimates of the FWER in the output for a 2-arm 3-stage trial, with I=D. To reproduce the simulation results presented in this paper, the design parameters can be amended for the desired specification.

```
nstage, nstage(2) alpha(0.25 0.1 0.025) omega(0.95 0.95 0.9) hr0(1 1) ///
hr1(0.75 0.75) accrue(500 500 500) arms(2 2) t(2 2) aratio(1) esb(peto)

nstage, nstage(2) alpha(0.25 0.1 0.025) omega(0.95 0.95 0.9) hr0(1 1) ///
hr1(0.75 0.75) accrue(500 500 500) arms(2 2) t(2 2) aratio(1) esb(obf)

nstage, nstage(2) alpha(0.25 0.1 0.025) omega(0.95 0.95 0.9) hr0(1 1) ///
hr1(0.75 0.75) accrue(500 500 500) arms(2 2) t(2 2) aratio(1) ///
esb(custom = 0.0005 0.001)
```

We also provide an example `nstage` command to identify the α_J which controls the FWER at 2.5% when incorporating a Haybittle-Peto early stopping rule, using the STAMPEDE trial from the results.

```
nstage, nstage(4) alpha(0.5 0.25 0.1 0.025) omega(0.95 0.95 0.95 0.9) ///
hr0(1 1) hr1(0.75 0.75) accrue(500 500 500 500) arms(6 6 6 6) t(2 4) ///
aratio(0.5) simcorr(250) corr(0.6) esb(peto) fwercontrol(0.025)
```

Appendix E Additional simulation study results

					Type I error rate				Power	
	α_1	α_2	Time S1	Time S2	No EB	With EB	Inflation	%	No EB	With EB
I=D, binding	0.5	0.025	1.54	3.12	0.0230	0.0233	0.0003	1%	0.8708	0.8710
	0.4	0.025	2.78	3.12	0.0231	0.0232	0.0001	0%	0.8743	0.8750
	0.3	0.025	2.04	3.12	0.0231	0.0231	0.0000	0%	0.8785	0.8784
	0.2	0.025	2.36	3.12	0.0233	0.0235	0.0002	1%	0.8842	0.8840
	0.1	0.025	2.82	3.12	0.0241	0.0242	0.0001	0%	0.8942	0.8946
I≠D, non-binding	0.5	0.025	0.91	3.12	0.0250	0.0255	0.0005	2%	0.8999	0.8998
	0.4	0.025	1.05	3.12	0.0250	0.0254	0.0006	2%	0.9005	0.8999
	0.3	0.025	1.20	3.12	0.0250	0.0254	0.0005	2%	0.9003	0.9001
	0.2	0.025	1.39	3.12	0.0250	0.0253	0.0006	2%	0.9004	0.8997
	0.1	0.025	1.65	3.12	0.0250	0.0252	0.0002	1%	0.9001	0.9002

Table 1: Impact of information time on the type I error rate with Peto efficacy boundary (EB) (p=0.0005). SEs all <0.0001. Lack-of-benefit boundary given by α_1, α_2. Allocation ratio=1.

			Type I error rate				Power	
	α_1	α_2	No EB	With EB	Inflation	%	No EB	With EB
I=D, binding	0.1	0.050	0.0500	0.0500	0.0000	0%	0.8999	0.8999
	0.1	0.025	0.0240	0.0240	0.0000	0%	0.8940	0.8940
	0.1	0.010	0.0093	0.0094	0.0001	1%	0.8869	0.8869
I≠D, non-binding	0.1	0.050	0.0500	0.0501	0.0001	0%	0.9001	0.9001
	0.1	0.025	0.0250	0.0254	0.0004	2%	0.9001	0.9001
	0.1	0.010	0.0100	0.0104	0.0004	4%	0.9001	0.9001

Table 2: Impact of the choice of the final stage significance level α_J on the type I error rate with Peto efficacy boundary (EB) (p=0.0005). SEs all <0.0001. Lack-of-benefit boundary given by α_1, α_2. Allocation ratio=1.

		Type I error rate				Power	
	Allocation Ratio	No EB	With EB	Inflation	%	No EB	With EB
I=D, binding	0.5	0.0240	0.0240	0.0000	0%	0.8944	0.8944
	0.6	0.0240	0.0240	0.0000	0%	0.8943	0.8943
	0.7	0.0240	0.0240	0.0000	0%	0.8942	0.8942
	0.8	0.0240	0.0240	0.0000	0%	0.8941	0.8941
	0.9	0.0240	0.0240	0.0000	0%	0.8941	0.8941
	1.0	0.0239	0.0239	0.0000	0%	0.8940	0.8940
I≠D, non-binding	0.5	0.0250	0.0253	0.0003	1%	0.9000	0.9000
	0.6	0.0250	0.0253	0.0003	1%	0.8999	0.8999
	0.7	0.0250	0.0253	0.0003	1%	0.8998	0.8998
	0.8	0.0250	0.0253	0.0003	1%	0.8999	0.8999
	0.9	0.0250	0.0252	0.0002	1%	0.8999	0.8999
	1.0	0.0250	0.0254	0.0004	2%	0.9000	0.9000

Table 3: Impact of the allocation ratio on the type I error rate with Peto efficacy boundary (EB) (p=0.0005). SEs all <0.0001. Lack-of-benefit boundaries $=0.1, 0.025$.

			FWER				Per-pair power		Any-pair power		All-pair power	
Comparisons		Stages	No EB	With EB	Inflation	%	No EB	With EB	No EB	With EB	No EB	With EB
I=D, binding	1	2	0.0239	0.0273	0.0034	14%	0.8940	0.8940	0.8940	0.8940	0.8940	0.8940
		3	0.0224	0.0261	0.0037	17%	0.8771	0.8771	0.8771	0.8771	0.8771	0.8771
		4	0.0213	0.0249	0.0036	17%	0.8553	0.8553	0.8553	0.8553	0.8553	0.8553
	2	2	0.0437	0.0495	0.0058	13%	0.8942	0.8942	0.965	0.965	0.8234	0.8234
		3	0.0410	0.0476	0.0066	16%	0.8773	0.8773	0.9575	0.9575	0.7971	0.7971
		4	0.0391	0.0455	0.0064	16%	0.8554	0.8554	0.9475	0.9475	0.7634	0.7634
	3	2	0.0605	0.0684	0.0079	13%	0.8941	0.8941	0.983	0.983	0.7705	0.7705
		3	0.0570	0.0658	0.0088	15%	0.8772	0.8772	0.9788	0.9788	0.738	0.738
		4	0.0543	0.0629	0.0086	16%	0.8554	0.8554	0.9731	0.9731	0.6971	0.6971
	4	2	0.0752	0.0846	0.0094	13%	0.8940	0.8940	0.9900	0.9900	0.7283	0.7283
		3	0.0708	0.0813	0.0105	15%	0.8769	0.8769	0.9873	0.9873	0.6912	0.6912
		4	0.0677	0.0781	0.0104	15%	0.8552	0.8552	0.9837	0.9837	0.6458	0.6458
	5	2	0.0882	0.0990	0.0108	12%	0.8939	0.8939	0.9934	0.9934	0.6934	0.6934
		3	0.0833	0.0956	0.0123	15%	0.8769	0.8769	0.9915	0.9915	0.6537	0.6537
		4	0.0798	0.0918	0.0120	15%	0.8553	0.8553	0.9891	0.9891	0.6049	0.6049
I≠D, non-binding	1	2	0.0250	0.0250	0.0000	0%	0.9001	0.9001	0.9001	0.9001	0.9001	0.9001
		3	0.0250	0.0250	0.0000	0%	0.9002	0.9002	0.9002	0.9002	0.9002	0.9002
		4	0.0250	0.0250	0.0000	0%	0.9001	0.9001	0.9001	0.9001	0.9001	0.9001
	2	2	0.0455	0.0455	0.0000	0%	0.9001	0.9001	0.9677	0.9677	0.8326	0.8326
		3	0.0455	0.0456	0.0001	0%	0.9002	0.9002	0.9676	0.9676	0.8327	0.8327
		4	0.0455	0.0455	0.0000	0%	0.9000	0.9000	0.9676	0.9676	0.8325	0.8325
	3	2	0.0628	0.0628	0.0000	0%	0.9001	0.9001	0.9845	0.9845	0.7818	0.7818
		3	0.0627	0.0627	0.0000	0%	0.9001	0.9001	0.9843	0.9843	0.7818	0.7818
		4	0.0627	0.0629	0.0002	0%	0.9001	0.9001	0.9845	0.9845	0.7816	0.7816
	4	2	0.0780	0.0780	0.0000	0%	0.9001	0.9001	0.9909	0.9909	0.7413	0.7413
		3	0.0780	0.0780	0.0000	0%	0.9000	0.9000	0.9909	0.9909	0.7412	0.7412
		4	0.0780	0.0781	0.0001	0%	0.9000	0.9000	0.9910	0.9910	0.7410	0.7410
	5	2	0.0916	0.0916	0.0000	0%	0.9000	0.9000	0.9941	0.9941	0.7076	0.7076
		3	0.0915	0.0915	0.0000	0%	0.9000	0.9000	0.9940	0.9940	0.7079	0.7079
		4	0.0915	0.0915	0.0000	0%	0.9000	0.9000	0.9941	0.9941	0.7076	0.7076

Table 4: Impact of the number of stages and arms on the max. FWER with an O'Brien-Fleming type efficacy boundary (EB). SEs all <0.0002. Lack-of-benefit boundaries as described in text. Allocation ratio=1.
